# A Challenging Diagnostic Process: A Case of Lewy Body Dementia in Long-Standing Schizophrenia

**DOI:** 10.7759/cureus.89967

**Published:** 2025-08-13

**Authors:** Aslam Iqbal, Mohamed Alarayedh, Tahir Iqbal, Samuel Olugbuyi, Claire Hicks-Walsh

**Affiliations:** 1 Psychiatry, Lincolnshire Partnership National Health Service (NHS) Foundation Trust, Lincoln, GBR

**Keywords:** antipsychotic adverse effects, antipsychotic-induced parkinsonism, diagnostic overshadowing, lewy body dementia, multi-disciplinary teams, overlapping features, schizophrenia

## Abstract

Lewy body dementia (LBD) is a progressive neurodegenerative disorder presenting with a wide range of cognitive, sleep, neuropsychiatric, motor, and autonomic symptoms. Diagnosing LBD in individuals with established psychiatric conditions, particularly chronic schizophrenia, presents significant challenges due to overlapping clinical features.

This case report outlines a case of a 78-year-old woman with a 48-year history of paranoid schizophrenia, who on her last admission exhibited new behavioural and functional decline. This included increased agitation, incoherent mumbling, visual hallucinations, self-harming behaviour, motor symptoms, and reduced responsiveness. Over time, she became increasingly frail and was displaying signs of extrapyramidal side effects on therapeutic doses of haloperidol.

Her presentation triggered regular comprehensive multidisciplinary team (MDT) discussions and reassessments, especially when the medical staff noted worsening motor symptoms, hypersensitivity to antipsychotics, and cognitive fluctuations. Although her primary psychiatric diagnosis had been schizophrenia for nearly five decades, the clinical evolution strongly indicated LBD, an emerging neurodegenerative process. Despite the absence of radiologic evidence, largely due to her condition, her new symptoms aligned with the diagnostic criteria for LBD. Subsequently, due to her poor physiological reserve, she was managed under a palliative care pathway.

This case highlights the importance of periodic diagnostic reassessment in a patient with a long-standing psychiatric disorder. Without careful review, there is a risk of diagnostic overshadowing, where new symptoms observed are miscredited to a historical diagnosis. Additionally, anchoring bias can further add to the issue as clinicians become fixated on the initial diagnosis. As a result, differentiating between chronic psychosis and evolving neurodegenerative conditions like LBD is crucial to avoid inaccurate management and to develop appropriate care plans, especially in vulnerable patients.

## Introduction

Lewy body dementia (LBD) is a late-onset neurodegenerative condition defined by intraneuronal inclusions of the proteins alpha-synuclein and ubiquitin in the brain stem, limbic area, forebrain, and neocortex [1]. This condition commonly features visual hallucinations, disrupted sleep, along with Parkinson-like movement issues such as tremors, muscle stiffness, and balance problems, within the first year of notable cognitive deficits. LBD patients may experience sudden shifts in alertness, slower thinking, visual-spatial difficulties, and challenges with verbal communication or decision-making [2]. To a lesser extent, other forms of hallucinations, depressive symptoms, and delusions may also be present. In later stages, physical problems such as swallowing issues and incontinence may emerge [1,2].

Schizophrenia is a mental disorder involving disturbances in many domains such as thoughts, perception, self-awareness, motivation, emotion, cognition, and behaviour. Core symptoms include persistent delusions, auditory hallucinations, thought disorder, and a sense of external control. Additionally, obvious impairments in social interaction, occupational, or personal functioning due to negative symptoms are also common features of schizophrenia. To a rarer extent, catatonia may also be present. Diagnosis requires symptoms to persist for at least one month that are not attributed to any other condition or substance [3]. 

Challenges in diagnosing LBD arise because both conditions present with overlapping symptomatology, which can often lead to overshadowing. However, they are distinguishable when evaluated in depth. The hallucinations typically experienced in LBD are visual and described in great detail, while in schizophrenia, patients typically experience third-person auditory hallucinations [1,3]. Moreover, LBD patients notably fluctuate in cognition, parkinsonism, and autonomic symptoms, whereas these features are less common in schizophrenia unless antipsychotic-induced [2,3]. A key feature is neuroleptic sensitivity, a core characteristic of LBD, which often manifests as worsening cognition, increased rigidity, and increased risk of neuroleptic malignant syndrome (NMS) when exposed to any antipsychotics [1]. Common examples of typical antipsychotics that are associated with NMS are haloperidol and fluphenazine [4].

## Case presentation

A 78-year-old female care home resident with a 48-year history of paranoid schizophrenia and mild learning disability was referred to the psychiatric Home Treatment Team (HTT) following an acute episode of self-harm. HTT comprises mental health professionals who review patients at their residence under the supervision of a consultant psychiatrist. The referral documented that she was striking herself repeatedly against furniture and would not engage with staff or peers. On initial assessment, the patient only intermittently responded to simple commands such as "open your eyes" or "squeeze my hand”. She was also seen responding to unseen stimuli and occasionally talking to herself. Therefore, HTT stopped her 5 mg olanzapine and commenced haloperidol 2 mg twice a day, aiming to gain rapid control of her agitation and psychosis. Additionally, 2 mg diazepam up to four times a day was prescribed to be used on a required basis.

Her psychiatric history obtained from clinical notes was notable for persistent auditory hallucinations and long-standing persecutory delusions, which had been managed over the years with different antipsychotics. In the year leading to this episode, the patient remained stable on olanzapine with no recent reports of psychotic relapse or self-harm. Her medical co-morbidities were significant and included Stage III chronic kidney disease, essential hypertension, gallstones with a dilated common bile duct, superior mesenteric artery thrombosis managed conservatively, dysphagia, and a history of total abdominal hysterectomy with bilateral salpingo-oophorectomy performed many years ago.

Despite the initial pharmacological interventions by HTT, there were no changes in her condition, and she continued to pose a significant risk to herself. For these reasons, she was detained and admitted under Section 2 of the UK Mental Health Act for assessment and treatment. Onward, management plans are drafted by the Multi-disciplinary Team (MDT) consisting of a consultant psychiatrist, resident doctors, physician associate, pharmacist, nurses, psychologist, care lead, occupational and physiotherapists.

An initial mental state examination (MSE) revealed that she was visibly distressed and fearful, with multiple bruises on her body. She frequently adopted a foetal position, avoided eye contact, and responded to unseen stimuli in short sentences or screaming. She managed to describe “small animals running around the room” or trying to pick out "worms" from her orifices. She did not engage in mood or cognitive assessments, and her insight and thought form were indeterminable due to her behaviours that continued to pose a risk to herself. Given the above evidence, the MDT decided to up-titrate haloperidol to 1.5 mg thrice daily. Soon after, the patient developed rigidity, hand tremors, and a shuffling gait, likely due to extrapyramidal side effects, prompting initiation of procyclidine 2.5 mg twice daily. However, within 10 days of increasing haloperidol, it was stopped alongside procyclidine due to worsening signs of neuroleptic sensitivity. Olanzapine was reintroduced up to 20 mg/day, as she had previously been stable on this medication. Lorazepam and promethazine were also prescribed intermittently to manage agitation and sleep. Despite these adjustments, she continued responding to visual hallucinations as evidenced by looking around the room, staring in a quizzical or fearful manner, giggling at times, most likely in line with thoughts, or auditory hallucinations as she was not congruent with the environment. She was unable to answer questions when asked repeatedly during MSE regarding her thoughts and perceptions, as her verbal communication was now limited to incoherent mumbling or silence. Her mobility deteriorated due to postural instability and orthostatic hypotension, resulting in several falls, requiring 1:1 supervision. Eventually, MDT decided to stop her olanzapine due to poor response, briefly improving her mobility and verbal engagement. However, this was short-lived, and after hospitalisation for suspected gastrointestinal bleeding, she returned to the ward with worsening faecal incontinence, abdominal discomfort, and further cognitive decline. Over the subsequent weeks, her clinical condition worsened as she could not engage in any conversation.

By day 77 of admission, the diagnostic picture became increasingly clear as the patient displayed core and supportive features of LBD. A formal diagnosis of LBD was made by the MDT. Table 1 below summarises the signs and symptoms considered by the MDT to reach this decision.

**Table 1:** Summary of clinical features displayed by patient throughout the admission REM: rapid eye movement

Category	Observed in patient	Supports LBD	Schizophrenia relapse
Cognitive decline	Progressive, with fluctuating attention and alertness	✅ Core LBD feature	❌ Atypical
Visual hallucinations	Well-formed and persistent (e.g., animals, worms)	✅ Core LBD feature	❌ Less typical or detailed
REM sleep disturbance	Acting out dreams	✅ Core LBD feature	❌ Not a primary feature
Motor signs	Rigidity, unsteady gait, parkinsonism	✅ Core LBD feature	❌ Rare
Neuroleptic sensitivity	Worsening rigidity, falls, confusion post-antipsychotic treatment	✅ Supportive LBD feature	❌ Rare
Autonomic dysfunction	Orthostatic hypotension and incontinence.	✅ Supportive LBD feature	❌ Atypical
Mood and behaviour changes	Agitation, inappropriate sexual behaviour, depression, anxiety	✅ Supportive LBD feature	🔄 Possible in both
Response to antipsychotics	Poor efficacy and decline with haloperidol and olanzapine	✅ Suggests neuroleptic sensitivity	❌ Historically stable on olanzapine
Speech/communication	Mumbling, incoherent, and reduced verbal engagement	✅ Supportive LBD feature	🔄 Incoherence overlaps with schizophrenia

Subsequent hospitalisation for rectal bleeding and gallstone disease further delayed the management of the new LBD diagnosis. On day 89 of admission, rivastigmine transdermal patches 4.6 mg/24 hrs were initiated for LBD management. Buprenorphine patch was introduced for the purpose of reducing pain without a sedative burden. Despite these robust interventions, her condition continued to decline as she became functionally bed-bound with profoundly limited oral intake and totally dependent for personal care. On day 104 of admission, MDT shifted her active treatment to a palliative approach. A few days later, the patient passed away on the ward, having received appropriate palliative care and comfort measures.

## Discussion

This case highlights the significant diagnostic challenges in distinguishing LBD from schizophrenia, mostly due to their overlapping features. However, with a comprehensive revision of several elements in this patient’s history and presentation, they provide strong evidence to diagnose LBD.

Diagnostic criteria for LBD are divided into core and additional clinical features [1]. Core features of LBD in this patient observed were progressive cognitive decline with variations in attention and alertness, well-formed and detailed visual hallucinations, acting out dreams during sleep and spontaneous motor signs consistent with parkinsonism, such as pronounced rigidity, and recurrent falls [1,5]. Supportive features were also documented as she experienced autonomic dysfunction such as orthostatic hypotension, mumbling and responding to auditory hallucination, behaviour supporting depression and anxiety, and striking sensitivity to neuroleptics [1]. The latter was demonstrated by profound physical and behavioural deterioration following administration of antipsychotics, namely olanzapine and haloperidol, where responses aligned with the well-documented adverse effects of these medications in LBD patients [6]. Initially, she was mobile, but due to disease progression, neuroleptic sensitivity, and declining physiological reserve, she became bed-bound. Her cognitive functioning was also progressively declining in a timely fashion during the admission, with rapidly fluctuating alertness. Throughout admission, she experienced fluctuating periods of lucidity which became increasingly shorter, less frequent, and unpredictable, until she was no longer able to communicate. The evidence above solidifies the LBD diagnosis made by the MDT [1,5].

Due to overlapping features, LBD can be misinterpreted as worsening psychosis. Without reassessment, clinicians might continue antipsychotics that are ineffective or even harmful. Up to 50% of LBD patients may exhibit neuroleptic sensitivity, leading to severe outcomes including parkinsonism, increased confusion, or death [1]. A schizophrenia relapse does not account for fluctuating alertness, rapid eye movement (REM) sleep disturbances, and persistent detailed visual hallucinations. In contrast, the worsening of motor symptoms, progressive cognitive decline, shorter lucid intervals, and heightened neuroleptic intolerance reinforced the likelihood of a neurodegenerative aetiology [1,3]. Though neuroimaging was not feasible due to her condition, clinical findings sufficiently supported the diagnosis under established criteria for LBD.

It has been reported that LBD takes significantly longer to diagnose than non-LBD dementia; 1.2 years vs. 0.6 years on average, respectively [7]. The overlap between long-standing psychiatric symptoms and emerging neurological signs increases the risk of diagnostic overshadowing, making timely re-evaluation critical when atypical features arise. This case also highlights the crucial role of MDT discussions in ensuring compassionate, holistic management across the disease spectrum from diagnosis to end-of-life care. Regular and focused interdisciplinary communication can help prevent delays in management and reduce the risk of diagnostic overshadowing [8].

## Conclusions

This case highlights the critical importance of clinical vigilance, flexibility in thinking, and team-based care in diagnosing LBD. When an older adult with a psychiatric history begins to deteriorate or shows unusual features of their existing diagnosis, it is vital to evaluate a broader differential to prevent further risk to the patient.
